# Personals

**Published:** 1916-03

**Authors:** 


### Personals.

Dr. R. A. Kooken, of Hamilton, has recently been very ill.

Dr. and Mrs. J. N. Fain have returned from Hot Springs, Ark.

Dr. Jones' family, of Glazier, have moved back to their country home.

Dr. Dallas, of Douglassville, is in Chicago attending medical lectures.

Dr. J. H. Walker, of Alvord, has returned from a business trip to Arkansas.

Dr. Rawlinson, of Hunnington, has returned from his studies at New Orleans.

Dr. Goodner, of Dublin, is able to be out again after an illness of three months.

Dr. and Mrs. B. F. Stevens, of El Paso, have returned from a trip to the Coast.

Dr. W. C. Mays, of Memphis, has moved his office to the rooms over Dowell and Howard.

Dr. and Mrs. Charles Taylor and daughter, of Sioux City, Iowa, are visiting in Beaumont.

Dr. and Mrs. C. N. Abbott, of McKinney, have recently spent a few weeks at Mineral Wells.

Dr. Rice, of Gainesville, is home from Fort Worth, where he underwent a successful operation.

Dr. Geo. W. Cross, of Eagle Lake, recently spent a month in New Orleans taking post-graduate work.

Dr. W. M. Fulbright of Ralls, went to Hagansport recently to attend his father who was quite ill of appendicitis.

Dr. W. T. Arnold, of Hemphill, is in New Orleans taking post-graduate courses in medicine at Tulane University.

Dr. R. P. Hudson, of Nashville, is a visitor in Brownsville, and may locate there for the practice of his profession.

Dr. F. P. Kennedy, of Brookshire, has recently taken a course of lectures in Tulane Medical College, New Orleans.

Dr. H. M. Phillips, of Kaufman, is in Chicago taking a special course in the treatment of the eye, ear, nose and throat.

Dr. and Mrs. W. H. Howell, who have been visiting in Crockett for several months, have returned to their home in Dallas.

Dr. P. G. Hays and family, of Bibb, have moved to Sipe Springs.

Dr. J. V. Guyton, of La Grange, has recently been in Smithville seeking a location for a sanitarium.

Dr. W. H. Grayson, of Bogart, recently had the misfortune to have an arm broken while cranking his car.

Dr. A. Suhler, of Waco, was the victim recently of an attack of paralysis. The attack affected his entire left side.

Dr. and Mrs. J. C. Henderson, of Waelder, have returned from San Antonio, where the doctor has been in a hospital.

Dr. N. E. Earrell has gone to the New Orleans Medical College to take a special course of the eye, ear, nose and throat.

Dr. J. D. Woolworth, of Keithville, La., and Miss Laura Flowers, were married at Henderson on Thursday of last week.

Dr. and Mrs. G. C. Hiatt, of Laredo, left recently for Chicago. The doctor has been ill for some time, and the trip is to consult a specialist.

Dr. M. H. E. Whiteside, of Timpson, left the 11th of February for a six weeks' stay in Marlin and New Orleans for the benefit of his health.

Dr. Dallas Southard, of Stamford, left recently for Chicago and Rochester to take a post-graduate course in special lines of medicine and surgery.

Dr. E. W. Davis and wife, who have been in Emory for several months, have gone to Texarkana, Texas, where he will enter the practice of his profession.

Dr. T. J. Crowe, of Dallas, attended the annual congress on medical education, public health and medical licensure, in Chicago, the first of February.

Dr. Gause W. Covington, of Fort Worth, has returned from Chicago, where he attended the lectures delivered by the famous specialist, Dr. Louis Schmidt.

Dr. W. N. Lemmon, of Greenville, who went to the Philippine Islands some years ago as a medical missionary for the Christian Church, will visit homefolks soon.

Dr. D. O. Lowry, of Cooper, who has been ill in Paris Sanitarium for some weeks, has undergone the third operation, and his condition is reported quite serious.

Dr. and Mrs. W. J. Hildebrand, of Gonzales, have returned from Baltimore, Md., where they accompanied the doctor's patient, Mr. W. B. Houston, several weeks ago.

A jury in the Federal court of Dallas recently found Dr. J. E. Baldwin guilty on five counts in an indictment charging him with violation of the Harrison anti-narcotic law.

Dr. John S. Turner, of Dallas, attended the meeting of the Council on Medical Education as the representative of the State Medical Association of Texas, in Chicago, the first of February.

Dr. R. J. Dice and son, Dewey, of Archer, have departed for the war zone of Europe, where the doctor will enter the army of the Allies as a physician and surgeon, and Dewey the hospital corps.

Dr. Arthur M. McElhannon, of Sherman, has recently been appointed by the Governor as a member of the State Board of Medical Examiners to fill the vacancy caused by the resignation of Dr. M. P. McElhannon, of Belton.

Dr. and Mrs. W. H. Bennett, of Falfurrias, have gone to New Orleans, where the doctor will take a post-graduate course in medicine and surgery. He will have access to practical work in surgery in the hospital where daily operations, more or less complicated, are performed.

Mrs. E. M. Maddux, woman physician, under a number of indictments for illegally issuing narcotic prescriptions, has withdrawn her appeal from the conviction in the only case on which she has been tried, and will serve out her sentence in jail. Mrs. Maddux was already in jail, having failed to make bond on five cases returned against her after the conviction was obtained. She was fined $25 and costs, and filed affidavit of her inability.

